# A Machine Learning-Based Prediction Model for Diabetic Kidney Disease in Korean Patients with Type 2 Diabetes Mellitus

**DOI:** 10.3390/jcm14062065

**Published:** 2025-03-18

**Authors:** Kyung Ae Lee, Jong Seung Kim, Yu Ji Kim, In Sun Goak, Heung Yong Jin, Seungyong Park, Hyejin Kang, Tae Sun Park

**Affiliations:** 1 Division of Endocrinology and Metabolism, Department of Internal Medicine, Jeonbuk National University Medical School, Research Institute of Clinical Medicine of Jeonbuk National University-Biomedical Research Institute of Jeonbuk National University Hospital, Jeonju 54907, Republic of Korea; kaleey@jbnu.ac.kr (K.A.L.);; 2 Department of Otorhinolaryngology-Head and Neck Surgery, Department of Medical Informatics, Jeonbuk National University Medical School, Research Institute of Clinical Medicine of Jeonbuk National University-Biomedical Research Institute of Jeonbuk National University Hospital, Jeonju 54907, Republic of Korea; kjsjdk@jbnu.ac.kr; 3 Department of Data Science, Evidnet, Seoul 06258, Republic of Koreahjk@evidnet.co.kr (H.K.)

**Keywords:** diabetic kidney disease, type 2 diabetes mellitus, prediction model, artificial intelligence, machine learning, risk prediction, clinical application

## Abstract

**Background/Objectives:** Diabetic kidney disease (DKD) is a major cause of end-stage kidney disease and a leading contributor to morbidity and mortality in patients with type 2 diabetes mellitus (T2DM). However, predictive models for DKD onset in Korean patients with T2DM remain underexplored. This study aimed to develop and validate a machine learning (ML)-based DKD prediction model for this population. **Methods:** This retrospective study utilized electronic health records from six secondary or tertiary hospitals in Korea. The Jeonbuk National University Hospital cohort was used for model development (ratio training: test data, 8:2), whereas datasets from five other hospitals supported external validation. We employed multiple ML algorithms, including lasso, ridge, and elastic net regression; random forest; XGBoost; support vector machines; and neural networks. The model incorporated demographic variables, comorbidities, medications, and laboratory test results. **Results:** Among 5120 patients with T2DM, 1361 (26.6%) developed DKD. In the development cohort, XGBoost achieved the highest predictive performance (AUC: 0.8099), followed by random forest and logistic regression models (AUCs: 0.7977–0.8019). External validation confirmed the model’s robustness with high AUCs (XGBoost: 0.8113, logistic regression models: 0.8228–0.8271). Key predictive factors included age; baseline estimated glomerular filtration rate; and creatinine, hemoglobin, and hemoglobin A1c levels. **Conclusions:** Our findings highlight the potential of ML-based approaches in predicting DKD in patients with T2DM. The superior performance of XGBoost and logistic regression models underscores their clinical utility. External validation supports the model’s generalizability. This model is a valuable tool for the early DKD risk assessment of Korean patients with T2DM.

## 1. Introduction

Type 2 diabetes mellitus (T2DM), characterized by chronic hyperglycemia and various metabolic abnormalities, is a major health problem worldwide [1], and the global prevalence of T2DM is increasing rapidly. In 2021, the International Diabetes Federation reported an estimated global DM prevalence of 10.5% (536.6 million people) among adults (aged 20–79 years). This prevalence is expected to increase to 12.2% (783.2 million people) by 2045 [2]. Korea has a high prevalence of DM, with one in seven individuals aged ≥30 years currently diagnosed with DM. The rise in T2DM prevalence is particularly notable [3].

Moreover, an estimated 700 million people worldwide have chronic kidney disease (CKD), and the prevalence and incidence of CKD have increased by 40% over the past 30 years [4,5]. DM and hypertension are the leading causes of CKD [5]. With the rapid increase in global T2DM prevalence, DM has become the most common cause of CKD and end-stage kidney disease (ESKD), contributing to increased morbidity and mortality [5]. According to an analysis of the Korean Renal Data System, a nationwide ESKD registry database managed by the Korean Society of Nephrology, DM is currently the leading cause of ESKD in Korea [6]. According to this registry, patients with diabetic kidney disease (DKD) undergoing dialysis have higher prevalence rates of cardiac and vascular diseases and experience more hospital admissions than non-diabetic CKD patients receiving dialysis [6]. According to recently updated epidemiological data on DKD in Korean patients with DM published by the Korean Diabetes Association, the estimated prevalence of DKD is 25.4% among individuals aged ≥30 years [7]. The prevalence of ESKD also continues to rise steadily, and atherosclerotic cardiovascular morbidity and mortality are significantly associated with the progression of DKD stages [7].

Approximately 20–40% of people with DM develop DKD [8]. This indicates that the risk of developing DKD varies among individuals. Therefore, the identification of high-risk patients and early intervention before the onset of DKD are important clinical issues. Numerous statistical studies have been conducted to predict the occurrence and progression of DKD [9,10,11,12,13]. Recently, studies using large-scale medical data to predict disease risk by using artificial intelligence (AI)-based machine learning (ML) and deep learning techniques have gained increased attention [14,15]. These approaches show considerable potential in accurately predicting DKD in patients from diverse backgrounds [16,17,18,19,20,21]. However, studies focusing on Korean populations are lacking.

In this study, we aimed to develop and validate an AI-based DKD prediction model using real-world data from electronic health records (EHRs) of secondary and tertiary medical institutions in Korea.

## 2. Methods

### 2.1. Study Design and Data Sources

This retrospective cohort study utilized the EHRs of Jeonbuk National University Hospital (JBUH), Ajou University Hospital (AUMC), Kyunghee University Hospital (KHMC), Kangwon National University Hospital (KWMC), Bucheon Sejong Hospital (Sejong-BCN), and Wonkwang University Hospital (WKUH) from January 2014 to December 2021. All EHR data were transformed into the Observational Medical Outcomes Partnership Common Data Model, a standardized database model that consolidates and integrates various real-world data sources, including EHRs [22]. The datasets provided comprehensive information on patients with DM, including data on demographics, disease diagnosis codes, laboratory test results, and medication prescriptions. Data from the JBUH were used to develop the model. Additionally, datasets from AUMC, KHMC, KWMC, Sejong-BCN, and WKUH were utilized for external validation.

### 2.2. Data Overview

#### 2.2.1. Data Extraction

Inclusion criteria were adults aged ≥19 years with T2DM who had at least two recorded serum creatinine tests. T2DM was defined by a code E11–E14 of the International Classification of Diseases, 10th Revision (ICD-10), or a prescription of at least one antidiabetic medication. Exclusion criteria were a diagnosis of DKD before the index date (ICD-10 codes E11.2–E14.2 or N08.3) or a diagnosis of another disease that may cause CKD (ICD-10 codes N18.x for CKD; N20–N21 for calculus of the kidney or urinary tract; Q61 for congenital renal disease; I12.0, I13.1, and I13.2 for hypertensive CKD; Z94.0 for kidney transplant status; K70–K74 for chronic liver disease; and C00–C97 for malignancy).

**Figure 1:**
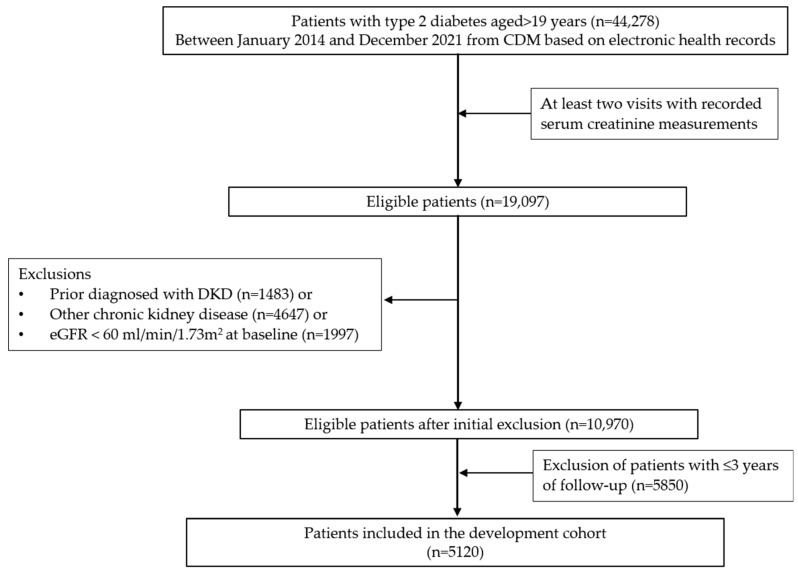
Flowchart depicting the selection process of the Jeonbuk National University Hospital cohort used for model development. CDM, common data model; DKD, diabetic kidney disease; eGFR, estimated glomerular filtration rate.

In the JBUH dataset, 44,278 adults aged ≥19 years were diagnosed with T2DM, of whom 19,097 had at least two serum creatinine measurements between January 2014 and December 2021. Of these, 1483 patients with a prior diagnosis of DKD, 4647 diagnosed with other forms of CKD, and 1997 with a baseline estimated glomerular filtration rate (eGFR) of ≤60 mL/min/1.73 m^2^ were excluded. Ultimately, 10,970 patients were considered eligible for analysis. Of these patients, only those with more than 3 years of follow-up were enrolled, resulting in a final cohort of 5120 patients in the JBUH dataset (Figure 1). For model training, the data were split in an 8:2 ratio, with 4096 and 1024 patients assigned to the training and test sets, respectively. External validation was performed using the AUMC, KHMC, KWMC, Sejong-BCN, and WKUH datasets. Patients with any missing data were excluded from the final analysis.

#### 2.2.2. Feature Selection and Preprocessing

The following clinical characteristics were considered for model development: age; sex; eGFR; creatinine, hemoglobin A1c (HbA1C), total cholesterol, high-density lipoprotein, and low-density lipoprotein levels; systolic and diastolic blood pressure; medical history, including hypertension, dyslipidemia, cardiac disease, and stroke; and medication prescriptions, including insulin, angiotensin-converting enzyme inhibitors, angiotensin receptor blockers, statins, and diuretics.

For laboratory tests, blood pressure, and medication prescriptions, we used data within the last 3 months before the index date; if two or more values existed, the value closest to the index date was selected. Complications were defined as the presence of at least one ICD-10 code or the use of medication corresponding to this disease.

For missing values in key variables, variables with ≤40% missing data were imputed using the mean, whereas variables with >40% missing values were excluded from the analysis. Excluded variables included body mass index, smoking and alcohol history, urine albumin to creatinine ratio, serum triglyceride, and uric acid.

Following feature selection, data were standardized to ensure that each variable had a mean of 0 and a standard deviation (SD) of 1. The models were trained on this normalized dataset using various ML algorithms.

### 2.3. ML Models

#### 2.3.1. Logistic Regression Models

Lasso, ridge, and elastic net regression analyses were performed to predict the occurrence of CKD. Logistic regression, a statistical method used to identify variables that affect a binary outcome (0 or 1), is the most commonly used analysis method in medical research. In our data, the large number of independent variables resulted in multicollinearity issues, with an increased risk of overfitting. To address these problems, we employed lasso and ridge regression models. These models, derived from statistical theory, are also widely applied in clinical prediction. These logistic models incorporate regularization to mitigate overfitting and enhance feature selection. We introduced elastic net regularization which admits the lasso and the ridge regression models as particular cases. The elastic net logistic regression attempts to maximize the penalized log-likelihood function defined as

$$l\left(\beta \right)-\lambda \left[\frac{\left(1-\alpha \right)}{2}\sum_{j=1}^{p}\beta_{j}^{2}+\alpha \sum_{j=1}^{p}\left|\beta_{j}\right|\right],$$

where $\beta ={\left(\beta_{1},\ldots ,\beta_{p}\right)}^{\prime }\in R^{p}$ is the regression coefficient vector, $l\left(\beta \right)$ is the log-likelihood function that one tries to maximize in the usual logistic regression without regularizing, λ>0 is a penalty parameter, and α∈[0,1] is a mixing parameter that balances between the lasso and the ridge. We note that α=0 and corresponds to the ridge, and α=1 corresponds to the lasso model. Elastic net combines the penalties of lasso and ridge regression, facilitating a more robust feature selection. The implementation was performed using the Python package “scikit-learn (version 1.4.2)” using predefined hyperparameters.

#### 2.3.2. Tree-Based Models

Random forest and eXtreme gradient boosting (XGBoost) are both tree-based models applied for non-linear classification. Random Forest employs bagging, an ensemble learning technique, by generating multiple decision trees using bootstrap sampling and randomly selecting subsets of features at each split, thereby improving predictive performance and reducing overfitting.

In contrast, boosting is another ensemble method that sequentially trains new decision trees, giving higher weights to misclassified instances to progressively enhance the model’s accuracy. XGBoost (version 2.1.1) is an advanced supervised learning algorithm that extends traditional gradient boosting by incorporating regularization techniques such as L1/L2 penalty, shrinkage, and early stopping to prevent overfitting. These enhancements improve computational efficiency while building stronger predictive models. Both models were optimized through a grid search to identify the best-performing hyperparameters.

#### 2.3.3. Support Vector Machines

Support vector machine (SVM) is a supervised learning method that performs classification and regression by finding a hyperplane in a high-dimensional or infinite-dimensional space. It determines the optimal decision boundary that separates two categories and predicts the category to which a new data point belongs. The fundamental idea of SVM is to maximize the margin between the decision boundary and the nearest data points, as a wider margin generally leads to better predictive performance. Support vector classification (SVC) is the classification version of SVM, which constructs a decision boundary that maximizes the margin of the given data.

#### 2.3.4. Deep Learning Models (Neural Network)

Neural networks are computational models inspired by the structure and function of the human brain, widely used for pattern recognition and predictive modeling. The multi-layer perceptron is a feedforward neural network architecture that comprises an input layer, one or more hidden layers, and an output layer, which consists of a single node for binary classification (CKD vs. non-CKD), using the sigmoid activation function. Each neuron in a layer is connected to neurons in the subsequent layer through weighted connections, and activation functions introduce non-linearity, enabling the model to learn complex relationships within the data. The multi-layer perceptron is particularly effective in supervised learning tasks, such as classification and regression, and is trained using backpropagation and gradient-based optimization techniques. In this study, we employed a multi-layer perceptron model to analyze and predict outcomes based on the given dataset, leveraging its capability to capture intricate patterns and interactions among features.

### 2.4. Hyperparameter Tuning

To ensure optimal model performance and generalizability, we conducted hyperparameter tuning using a grid search approach for all machine learning models. Key parameters were systematically explored to identify the best configuration.

For lasso, elastic net and ridge regression, regularization strengths were tested over a wide range (0.0001–10,000). In RF, the number of trees (50–150), maximum depth (4–8), and feature selection ratio (0.4–0.8) were optimized. XGBoost was tuned for learning rate (0.01–0.2), number of estimators (50–150), and tree depth (4–8), along with regularization parameters like gamma. SVC was optimized for C (0.001–100) and gamma (0.001–100). Lastly, for the neural network, we tuned alpha (0.001–10) and learning rate (0.0001–0.2). These hyperparameter tuning efforts contributed to improving model accuracy and reducing overfitting.

### 2.5. Model Evaluation

Model performance was primarily evaluated using the area under the receiver operating characteristic curve as the primary metric. Additionally, to provide a more comprehensive and intuitive assessment, we analyzed sensitivity, specificity, accuracy, and F1-score for each model. A confusion matrix was also constructed to illustrate classification performance in terms of true positives, true negatives, false positives, and false negatives (Supplementary Figure S1). Internal validation was performed using the JBUH test set, whereas external validation was performed using the AUMC, KHMC, KWMC, SEJONG-BCN, and WKUH datasets.

## 3. Results

### 3.1. Demographic Characteristics of Patients from JBUH, AUMC, KHMC, KWMC, Sejong-BCN, and WKUH

The JBUH dataset included 5120 patients with T2DM, of whom 1361 (approximately 27%) developed DKD during the observation period. The mean age of the participants was 61.5 (SD: 12.4) years, with 2125 (42%) being men. In the external validation cohort, the number of patients with T2DM and the number of those who developed DKD (%) in each hospital were as follows: AUMC (717 patients with T2DM, of whom 95 [13%] developed DKD), KHMC (520 patients, of whom 237 [46%] developed DKD), KWMC (969 patients, of whom 222 [23%] developed DKD), Sejong-BCN (1280 patients, of whom 232 [18%] developed DKD), and WKUH (707 patients, of whom 161 [23%] developed DKD). Overall, the external validation set included 4193 patients with T2DM, of whom 947 (23%) developed DKD. The mean age of the participants was 60.2 (SD: 12.6) years, with 1633 (39%) being men. Table 1 and Table 2 present the demographic data of the development and external validation cohorts according to DKD status, respectively.

### 3.2. Comparisons of Prediction Model Performance

**Figure 2:**
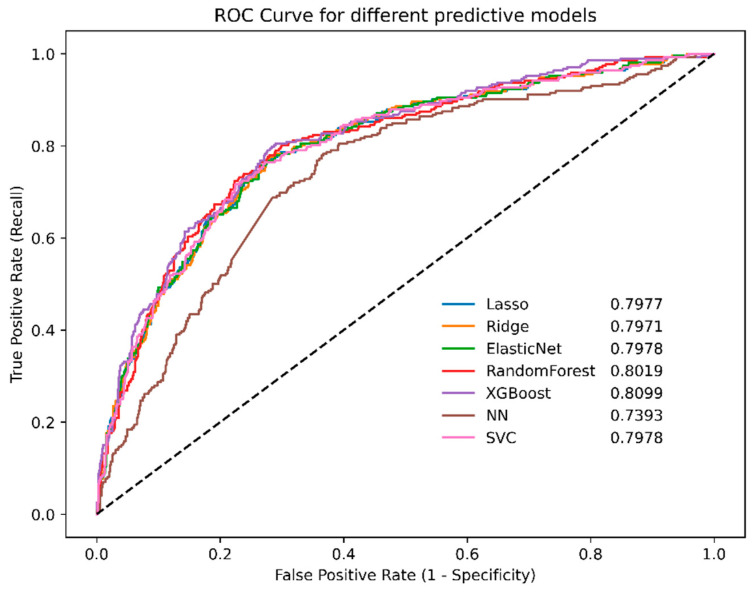
ROC curves for different machine learning models in the Jeonbuk National University Hospital cohort. NN, neural network; ROC, receiver operating characteristic; SVC, support vector machines; XGBoost, eXtreme gradient boosting.

The highest-performing model was XGBoost, exhibiting an area under the receiver operating characteristic curve of 0.8099 (Figure 2). The other models yielded the following area under the curve (AUC) values: random forest, 0.8019; ridge logistic regression, 0.7971; lasso logistic regression, 0.7977; and elastic net logistic regression, 0.7978.

**Figure 3:**
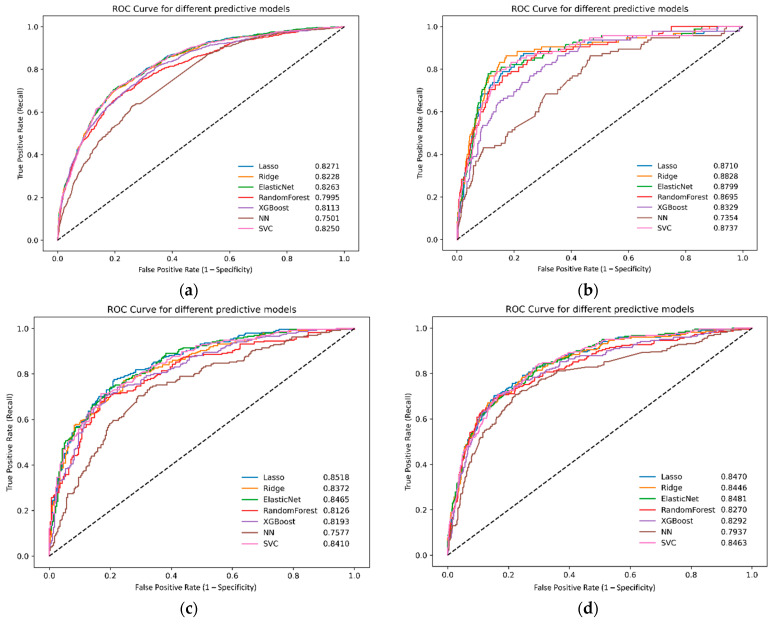

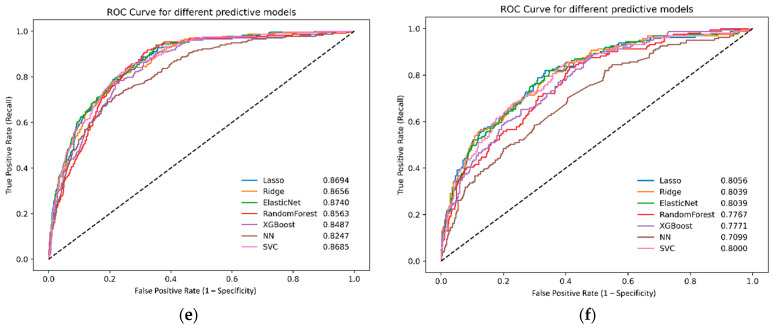
Receiver operating characteristic curves for different machine learning models in the external validation dataset: (**a**) all databases, (**b**) AUMC, (**c**) KHMC, (**d**) KWMC, (**e**) Sejong-BCN, and (**f**) WKUH. NN, neural network; ROC, receiver operating characteristic; SVC, support vector machines; XGBoost, eXtreme gradient boosting; AUMC, Ajou University Hospital; KHMC, Kyunghee University Hospital; KWMC, Kangwon National University Hospital; Sejong-BCN, Bucheon Sejong Hospital; WKUH, Wonkwang University Hospital.

External validation using the AUMC, KHMC, KWMC, Sejong-BCN, and WKUH datasets revealed similar trends. The XGBoost model maintained strong performance, achieving an AUC of 0.8113. The lasso logistic regression model maintained strong performance, with an AUC of 0.8271. The elastic net and ridge logistic regression models achieved AUC values of 0.8263 and 0.8228, respectively (Figure 3). The SVM model also demonstrated good performance, with an AUC of 0.8250.

### 3.3. Other Performance Metrics of Each Machine Learning Models

To comprehensively evaluate model performance beyond the area under the receiver operating characteristic curve (AUC), we analyzed additional metrics, including sensitivity, specificity, accuracy, precision, and F1 score (Table 3).

Among the models, XGBoost achieved the highest AUC (0.8099), demonstrating a balanced performance with a sensitivity of 0.7316 and specificity of 0.7566. RF exhibited the highest sensitivity (0.7647), indicating a strong ability to identify positive cases, but at the cost of a lower specificity (0.7354). Conversely, lasso regression showed the highest specificity (0.7726), suggesting a better ability to exclude false positives, but with a relatively lower sensitivity (0.6985).

In terms of overall accuracy, all models performed similarly, ranging between 0.7432 and 0.7529, with F1 scores ranging from 0.5969 to 0.6127. These results indicate that, although different models have varying strengths in detecting true positives versus true negatives, XGBoost provided the most well-balanced classification performance.

### 3.4. Feature Importance Analysis

**Figure 4:**
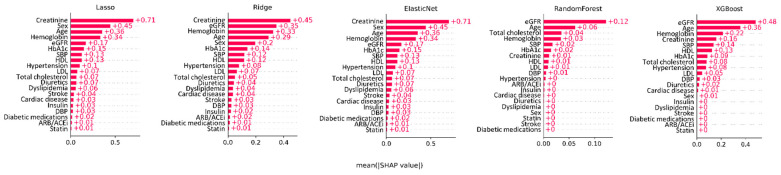
SHapley Additive exPlanation values showing feature importance in various machine learning models. DBP, diastolic blood pressure; eGFR, estimated glomerular filtration rate; HbA1C, hemoglobin A1c; HDL, high-density lipoprotein; LDL, low-density lipoprotein; SBP, systolic blood pressure; XGBoost, eXtreme gradient boosting.

Feature importance analysis across all models demonstrated that eGFR, creatinine, age, hemoglobin, and HbA1C were the most critical variables in predicting CKD in patients with DM (Figure 4). In the logistic regression models (lasso, ridge, and elastic net), the variable ranking was as follows: creatinine, sex, age, hemoglobin, eGFR, and HbA1c contributed the most to the model’s predictions. In the tree-based models (random forest and XGBoost), the ranking of variables was consistent, with age and eGFR contributing the most to the model’s predictions.

## 4. Discussion

This study applied AI to develop a DKD prediction model using real-world medical data from patients treated at a tertiary medical institution in South Korea. The model was externally validated using datasets from five referral institutions, demonstrating strong performance. Only a few studies have aimed at predicting the first occurrence of DKD, and none of these studies focused on Korean patients. Due to the low rate of albuminuria testing in real-world practice, our model, which incorporates demographic and laboratory variables, offers a practical alternative for early DKD risk assessment. Its integration into EHR systems may facilitate automated risk stratification, thereby enabling timely interventions.

DKD, a major microvascular complication of DM, is a leading cause of ESKD, cardiovascular disease, and all-cause mortality in individuals with DM [23]. The global incidence of DKD among people with T2DM increased by 74% from 1990 to 2017 [24], and South Korea reported the highest annual average growth rate of DM-related ESKD treatment from 2010 to 2020 [24]. This significant increase in DKD prevalence is expected to not only increase the morbidity and mortality among patients with DM but also reduce their quality of life and increase the health burden.

The risk factors for DKD include both modifiable (high blood glucose, high blood pressure, blood lipid abnormalities, insulin resistance, metabolic syndrome, obesity, and smoking) and non-modifiable (increasing age, young-onset DM, prolonged DM duration, genetic factors, ethnicity, and family history of DKD) factors [23]. Although older age is a key risk factor, individuals diagnosed with DM at a young age are also at a high risk of developing DKD. In recent years, the number of patients with young-onset T2DM who have obesity and metabolic syndrome components has been increasing in many countries, including Korea. These patients frequently have multiple risk factors for developing DKD [25]. According to the 2024 Diabetes Fact Sheet by the Korean Diabetes Association, 35% of young patients with DM have hypertension, 75% have hypercholesterolemia, and 3 out of 10 have both conditions. Nearly 9 out of 10 patients (87%) have obesity, with a body mass index of ≥25 kg/m^2^ [26]. Patients who developed DKD experienced various complications, including hypertension, dyslipidemia, and abdominal obesity, with poorer control rates compared to those who did not develop DKD [7].

Despite the well-known risk factors mentioned above, predicting the onset of DKD in patients with DM remains challenging. DKD develops in approximately half of patients with DM [8]. Due to limited medical resources, treating all patients with DM equally to prevent DKD is not considered cost-effective. Therefore, an approach is needed to appropriately stratify the risk for each patient and provide early intervention to those at high risk of developing DKD.

Albuminuria is an established biomarker of DKD progression. However, predicting the onset of DKD prior to renal damage remains challenging, as albuminuria is typically detected in the urine after glomerular damage has already progressed. Furthermore, albuminuria can be influenced by various factors, including fever, vigorous exercise, hyperglycemia, and hypertension, necessitating repeat testing and leading to low performance in actual clinical practice [27]. In some patients with DM, the decline in glomerular filtration rate progresses without preceding proteinuria [28]. For these reasons, diabetes practice guidelines recommend annual urine albumin-to-creatinine ratio and GFR tests, but early identification of high-risk patients remains challenging. Consequently, not only numerous preclinical studies have been conducted to identify biomarkers capable of early predicting DKD [29] but also clinical studies utilizing available clinical information to predict DKD [9,10,16,17,30].

Regression analysis is a useful statistical method for the development of prediction models and the identification of contributing factors associated with DKD [31]. Depending on the nature of the dependent variables, linear, logistic, and survival regression analyses can be used. The effects of independent variables on the dependent variable can be evaluated using regression coefficients, making this method valuable in the prediction of DKD [31]. As a logistic model like, lasso is relatively simple; nonetheless, it achieved a comparatively high AUC (0.7977) and the highest specificity (0.7726), making it a good candidate for cases where minimizing false positives is crucial, such as screening tests requiring high specificity or when model interpretability is a priority.

In the era of big data, ML, a branch of AI, is expected to develop predictive models that outperform traditional statistical methods [31]. ML methods are employed to analyze large datasets, utilizing computer algorithms to identify data patterns and relationships, which are subsequently used to predict new data.

Recently, numerous ML-based DKD prediction models have been developed, with most demonstrating satisfactory performance and suggesting potential clinical applicability [16,17,18,19,20,21]. However, most of these studies did not include Korean populations and focused on predicting disease progression in patients with pre-existing DKD. No AI-based studies have investigated the prediction of DKD occurrence in Korean patients with DM exhibiting normal renal function except for a few studies using statistical methods [10,32,33]. To date, AI-based prediction models have been developed in Korea to predict acute kidney injury in patients with trauma or transplant recipients or to predict acute rejection after kidney transplantation [34,35,36]. The present study demonstrates that an AI-based prediction model can exhibit excellent performance and high clinical applicability for the occurrence of DKD in the Korean population. Applying this prediction model together with proteinuria and glomerular filtration rate tests can select patients at high risk of developing DKD and reduce the risk through early and aggressive therapeutic intervention.

The performance variations across different models can be attributed to their underlying mechanisms and how they handle feature interactions, regularization, and complexity.

RF demonstrated the highest sensitivity (0.7647) among all the models. This can be explained by its ensemble learning approach, where multiple decision trees are trained on random subsets of the data with feature bagging. The aggregation of diverse trees helps capture complex patterns, particularly in identifying positive cases, leading to a higher true positive rate. However, RF also exhibited a slightly lower specificity (0.7354) compared to other models. This is likely because RF tends to reduce variance at the cost of increased false positives, as it focuses on minimizing classification errors by leveraging majority voting. The lower specificity suggests that RF may overpredict positive cases, which can be beneficial in scenarios where false negatives are more critical than false positives.

XGBoost, in contrast, achieved the highest AUC (0.8099) and demonstrated balanced sensitivity (0.7316) and specificity (0.7566), making it the most well-rounded model in terms of classification performance. The strength of XGBoost lies in its gradient boosting framework, which sequentially builds decision trees where each new tree corrects the errors of the previous one. This iterative learning process, combined with regularization techniques such as L1/L2 penalties, column sampling, and shrinkage, prevents overfitting while improving generalization. Additionally, XGBoost’s ability to handle complex feature interactions efficiently enables it to achieve strong performance across different evaluation metrics.

The feature importance analysis conducted in our study identified well-established risk factors, such as age, baseline renal function, and HbA1C level (which reflects blood sugar control status), as major variables contributing to the prediction model. Additionally, the hemoglobin level was recognized as an important variable across several models. Other machine learning-based studies also suggest that hemoglobin is among the most influential variables in CKD prediction [37]. Anemia, a common consequence of CKD, is increasingly recognized as a non-canonical risk factor for CKD progression [37]. It induces renal hemodynamic alterations and tissue hypoxia, with chronic hypoxia being a key driver of tubulointerstitial damage [38]. As it is a test that can be easily performed in clinical practice, it should be considered along with other key variables in predicting DKD in clinical practice.

For patients identified as being at a high risk of developing DKD according to the predictive model, active risk factor management and therapeutic approaches are essential. The primary treatment strategy for DKD is prevention through glycemic control and the management of other modifiable risk factors. Once DKD develops, renin–angiotensin system inhibitors are one of the standard treatments. Recent randomized controlled trials have demonstrated that sodium-glucose cotransporter-2 inhibitors can delay the progression of DKD independently of their glucose-lowering effect [39]. Moreover, semaglutide, a novel glucagon-like peptide-1 receptor agonist, has significant effects in delaying the progression of DKD [40]. In addition, finerenone, a nonsteroidal mineralocorticoid antagonist with anti-inflammatory and antioxidant effects, provides additional clinical benefits in patients using sodium-glucose cotransporter-2 and renin–angiotensin system inhibitors as a background treatment [41]. A meta-analysis revealed that the combination of these three medications had beneficial effects in slowing the progression of DKD [42]. Considering the availability and cost of these medications, the recommendation of prescribing these drugs to all patients is not a cost-effective approach. Therefore, the identification of high-risk patients is crucial.

This study has certain limitations. First, as a retrospective study, a considerable amount of data was missing. Key DKD predictors had high rates of missing data (e.g., the albumin-to-creatinine ratio and body mass index were available in approximately 10% and 30% of cases, respectively), making them inapplicable to the model. This aligns with previous studies reporting low rates of proteinuria screening due to factors like lack of patient compliance [42]. Additionally, essential medical data—such as DM duration, family history of DKD, alcohol consumption, and smoking history—exist as unstructured records in hospital systems, preventing their conversion into a common data model (CDM). To develop a generally applicable and high-performance prediction model, constructing a comprehensive dataset of patients with diabetes with diverse characteristics is of utmost importance. We plan to further expand the CDM dataset of patients with DM through prospective data collection, which is expected to further expand and validate our prediction model. Second, since this study was conducted in tertiary referral centers, the patient population may not fully represent primary care settings, where DKD risk profiles may differ. Future studies should validate our model in diverse healthcare environments to ensure broader applicability. Third, the complexity and computational cost of certain machine learning models must be considered. RF and XGBoost, although achieving strong predictive performance, suffer from interpretability issues due to their ensemble nature, making it difficult to extract intuitive insights. Additionally, RF requires extensive computational resources due to the need for training multiple trees, while XGBoost has high memory consumption, particularly when handling large datasets. XGBoost is also sensitive to outliers, which may impact its robustness with noisy data. Meanwhile, simpler models such as lasso, ridge, and elastic net offer better interpretability but may have limitations in capturing complex nonlinear relationships. Fourth, although our study evaluated feature importance, future work should focus on improving the explainability of our AI model to build clinician trust. Using SHapley Additive exPlanations values and feature importance visualizations can help clinicians interpret model predictions, ensuring transparency and reliability in decision-making.

Despite these shortcomings, the significance of this study lies in the development of a robust prediction model utilizing easily accessible clinical variables, based on a large, real-world cohort of Korean patients with diverse characteristics who were followed up for more than 3 years.

In conclusion, we developed an AI-based model to predict the occurrence of DKD in Korean patients with T2DM and validated the model’s performance using data from patients with DM across five referral hospitals. Key predictors of DKD in our model include older age, male sex, eGFR, hyperglycemia, and anemia. Given the limitations of albuminuria testing in routine practice, our AI-driven model provides a more accessible tool for early risk assessment, facilitating timely clinical interventions.

## Figures and Tables

**Table 1:** Demographic characteristics of the development cohort (Jeonbuk National University Hospital).

Feature	Overall (n = 5120)	No DKD (n = 3759)	DKD (n = 1361)
Sex (male), n (%)	2125 (42)	1521 (40)	604 (44)
Age (years), mean (SD)	61.53 (12.42)	59.16 (12.11)	68.09 (10.82)
Systolic blood pressure (mmHg), mean (SD)	137.87 (22.44)	137.12 (22.16)	139.83 (23.05)
Diastolic blood pressure (mmHg), mean (SD)	85.52 (15.35)	85.49 (15.32)	85.60 (15.45)
Hypertension, n (%)	859 (17)	550 (15)	309 (23)
Dyslipidemia, n (%)	509 (10)	383 (10)	126 (9)
Cardiac diseases, n (%)	1175 (23)	776 (21)	399 (29)
Stroke, n (%)	380 (7)	260 (7)	120 (9)
Insulin, n (%)	725 (14)	517 (14)	208 (15)
ARB or ACEi, n (%)	741 (14)	517 (14)	224 (16)
Diuretics, n (%)	349 (7)	218 (6)	131 (10)
Statin, n (%)	1162 (23)	835 (22)	327 (24)
Creatinine (mg/dL), mean (SD)	0.75 (0.20)	0.72 (0.19)	0.82 (0.20)
eGFR mL/min/1.73 m^2^, mean (SD)	95.30 (16.81)	98.97 (15.95)	85.16 (14.85)
HbA1c, mg/dL (%), mean (SD)	7.41 (1.56)	7.41 (1.55)	7.42 (1.61)
Hemoglobin g/dL, mean (SD)	13.70 (1.90)	13.93 (1.84)	13.05 (1.92)

ACEi, angiotensin-converting enzyme inhibitor; ARB, angiotensin receptor blocker; HbA1c, hemoglobin A1c; SD, standard deviation; eGFR, estimated glomerular filtration rate; DKD, diabetic kidney disease.

**Table 2:** Demographic characteristics of the external validation cohort (AUMC, KHMC, KWMC, Sejong-BCN, and WKUH).

Feature	Overall (n = 4193)	No DKD (n = 3246)	DKD (n = 947)
Sex (male), n (%)	1633 (39)	1255 (0.39)	378 (40)
Age (years), mean (SD)	60.16 (12.58)	57.88 (12.10)	67.99 (10.97)
Systolic blood pressure (mmHg), mean (SD)	134.65 (19.86)	133.52 (19.44)	138.50 (20.76)
Diastolic blood pressure (mmHg), mean (SD)	81.88 (12.06)	81.71 (11.99)	82.44 (12.30)
Hypertension, n (%)	1471 (35)	1027 (32)	444 (47)
Dyslipidemia, n (%)	1076 (26)	771 (24)	305 (32)
Cardiac diseases, n (%)	1895 (45)	1325 (41)	570 (60)
Stroke, n (%)	476 (11)	305 (9)	171 (18)
Insulin, n (%)	143 (3)	105 (3)	38 (4)
ARB or ACEi, n (%)	1044 (25)	752 (23)	292 (31)
Diuretics, n (%)	413 (10)	273 (8)	140 (15)
Statin, n (%)	413 (10)	273 (8)	140 (15)
Creatinine, mg/dL, mean (SD)	0.80 (0.18)	0.78 (0.17)	0.87 (0.18)
eGFR mL/min/1.73 m^2^, mean (SD)	93.64 (16.60)	96.98 (15.73)	82.20 (14.28)
HbA1c mg/dL (%), mean (SD)	6.74 (1.45)	6.76 (1.46)	6.70 (1.45)
Hemoglobin, g/dL, mean (SD)	14.09 (1.71)	14.24 (1.67)	13.61 (1.76)

ACEi, angiotensin-converting enzyme inhibitor; ARB, angiotensin receptor blocker; HbA1c, hemoglobin A1c; SD, standard deviation; eGFR, estimated glomerular filtration rate; DKD, diabetic kidney disease;

**Table 3:** AUC ROC and other performance metrics of each machine learning model.

Model	AUC ROC	Sensitivity	Specificity	Accuracy	Precision	F1 Score
Lasso	0.7977	0.6985	0.7726	0.7529	0.5263	0.6003
Ridge	0.7971	0.7022	0.7646	0.7480	0.5190	0.5969
Elastic Net	0.7978	0.7022	0.7673	0.7500	0.5219	0.5988
Random Forest	0.8019	0.7647	0.7354	0.7432	0.5111	0.6127
XGBoost	0.8099	0.7316	0.7566	0.7500	0.5209	0.6085
Neural Network	0.7393	0.4081	0.8564	0.7373	0.5068	0.4521
SVC	0.7978	0.7243	0.7566	0.7480	0.5184	0.6043

## Data Availability

The raw data supporting the conclusions of this article are available from the authors upon reasonable request.

## Supplementary Materials

The following supporting information can be downloaded at: https://www.mdpi.com/article/10.3390/jcm14062065/s1, Figure S1: Confusion matrices showing true positives, true negatives, false positives, and false negatives identified by the different machine learning models in the Jeonbuk National University Hospital cohort.

## Abbreviations

The following abbreviations are used in this manuscript:

AI	artificial intelligence
AUC	area under the curve
AUMC	Ajou University Hospital
CDM	common data model
CKD	chronic kidney disease
DKD	diabetic kidney disease
eGFR	estimated glomerular filtration rate
EHR	electronic health record
ESKD	end-stage kidney disease
HbA1C	hemoglobin A1c
JBUH	Jeonbuk National University Hospital
KHMC	Kyunghee University Hospital
KWMC	Kangwon National University Hospital
ML	machine learning
SD	standard deviation
Sejong-BCN	Bucheon Sejong Hospital
SVM	support vector machine
T2DM	type 2 diabetes mellitus
WKUH	Wonkwang University Hospital
XGBoost	eXtreme Gradient Boosting
