# Supplementary material for: A Machine Learning-Based Prediction Model for Diabetic Kidney Disease in Korean Patients with Type 2 Diabetes Mellitus
Source: J Clin Med. 2025 Mar 18;14(6):2065. doi: 10.3390/jcm14062065 (PMC11942948; doi:10.3390/jcm14062065)
Supplement: Supplementary file 1 [file jcm-14-02065-s001.zip › jcm-3485624-supplementary.pdf]

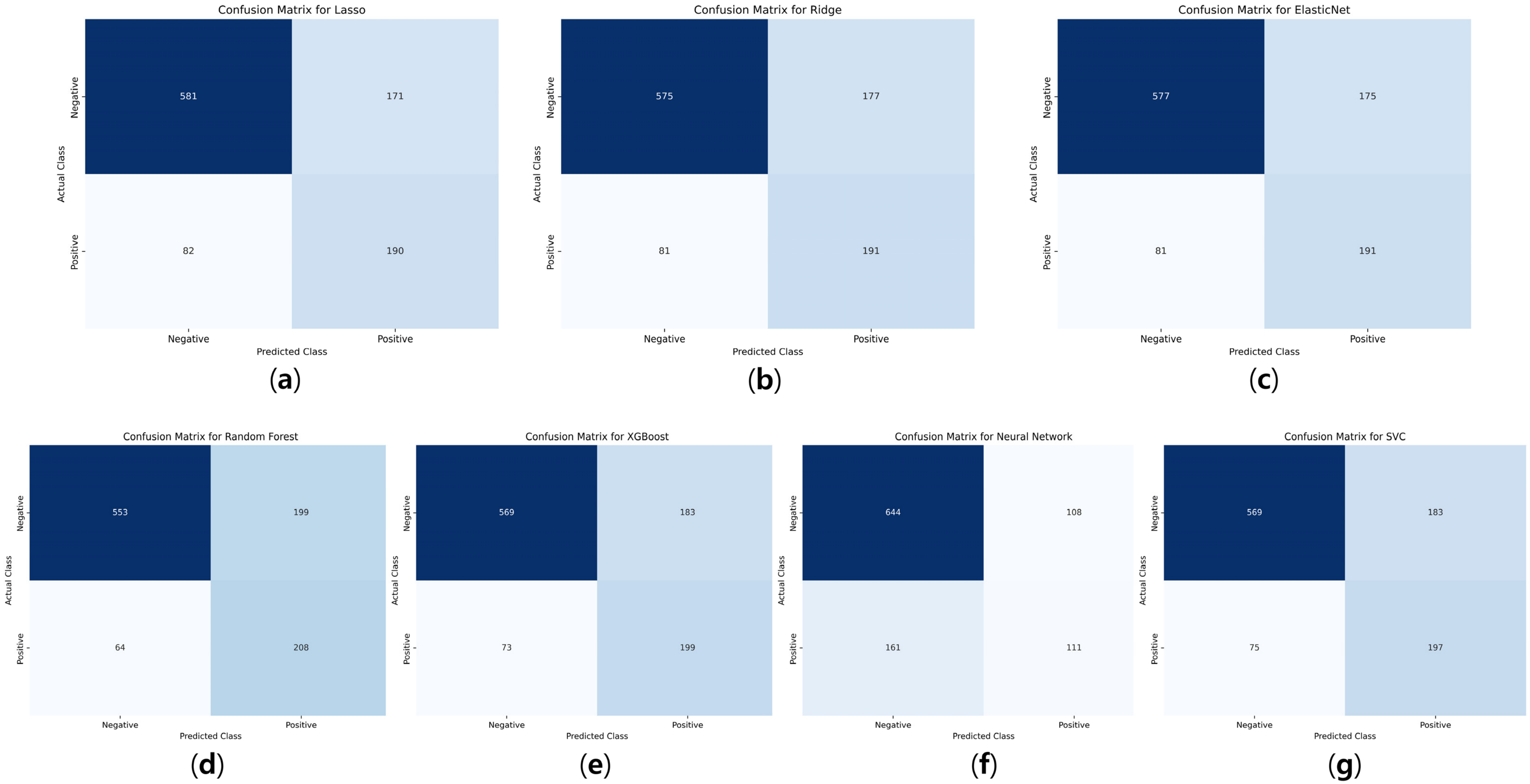

Scheme 1. Confusion matrices showing true positives, true negatives, false positives, and false negatives identified by the different machine learning models in the Jeonbuk National University Hospital cohort.
